# The kinetic requirements of extreme qPCR

**DOI:** 10.1016/j.bdq.2019.100081

**Published:** 2019-03-13

**Authors:** Adam L. Millington, Jessica A. Houskeeper, John F. Quackenbush, James M. Trauba, Carl T. Wittwer

**Affiliations:** Department of Pathology, University of Utah Health Sciences Center, Salt Lake City, UT, 84108, United States

**Keywords:** HSM, high-speed melting, Cq, quantification cycle, T_m_, melting temperature, SNV, single nucleotide variants, Extreme PCR, High speed melting, Microfluidics, PCR kinetics

## Abstract

The kinetic requirements of quantitative PCR were experimentally dissected into the stages of DNA denaturation, primer annealing, and polymerase extension. The temperature/time conditions for 2 stages were kept optimal, while the other was limited until the amplification efficiency decreased as measured by an increase in quantification cycle (Cq). Extension was studied in a commercial capillary LightCycler®. Using a rapid deletion mutant of Taq (KlenTaq^™^), about 1 s was required for every 70 bp of product length. To study annealing and denaturation times of <1 s, a custom “extreme” PCR instrument with 3 temperatures was used along with increased primer and polymerase concentrations. Actual sample temperatures and times were measured rather than programmed or predicted. For denaturation, 200–500 ms above the denaturation threshold was necessary for maximal efficiency. For annealing, 300-1000 ms below the annealing threshold was required. Temperature thresholds were set at 98% primer annealing or PCR product denaturation as determined experimentally by melting curves. Progressing from rapid cycle PCR to extreme PCR decreased cycling times by 10–60 fold. If temperatures are controlled accurately and flexibility in reagents is allowed, PCR of short products can be performed in less than 15 s. We also put PCR in context to other emerging methods and consider its relevance to the evolution of molecular diagnostics.

## 1 Introduction

As one of the simplest and most direct molecular tools available, PCR continues as a dominant force in molecular diagnostics. However, our understanding of how such a basic process works lags behind our enthusiasm to use it. For example, the kinetic limits of PCR remain controversial and misunderstood. There is a mismatch between common instrumentation and the kinetic potential of PCR. Quantitative PCR and melting analysis can be performed in less than a minute [1], but most commercial protocols recommend over an hour. PCR speeds have increased over the years, although most instruments today remain at slower, legacy speeds. Comparative speeds are best defined directly, either in the time for 1 cycle, or the total time for 30 cycles (Table 1).

**Table 1:** Polymerase Chain Reaction (PCR) Speeds.

**PCR Speed**	**Year of Introduction**	**Time for 30 Cycles**	**Time for 1 Cycle**
Legacy	1988	2.25 hours	4.5 min
Rapid Cycle	1991	10-30 min	20-60 s
Fast	2000s	30-60 min	1-2 min
Ultra-Fast	2010s	2-10 min	4-20 s
Extreme	2015	15-60 s	0.5-2 s

Legacy PCR with heat stable Taq polymerase was introduced in 1988 and called for 2–2.5 h for 25–35 cycles [2]. The instruments were so slow that about 90% of the time was spent ramping between temperatures. Rapid cycle PCR in capillaries soon followed, requiring only 10–30 min for 30 cycles [3,4]. Various fast techniques based on proprietary reagents were developed in the 2000s, although these were typically slower (30–60 min) than rapid cycle PCR and used conventional instruments. Ultra-fast techniques used a multitude of creative instruments in the 2010s with PCR completion typically in 2–10 min [5]. Finally, by increasing concentrations of primers and rapid polymerases and using extreme temperature cycles of 0.5–2 s, robust PCR amplifications were obtained in 15–60 s without sacrificing sensitivity, specificity, efficiency, or yield. Such extreme speeds, originally obtained with bulk sample transfer between water baths [6], have now been replicated on at least three different microfluidic platforms [1,7,8].

All extreme PCR instruments reported to date rely on two-temperature cycling—that is, the primer annealing and polymerase extension stages are combined and performed simultaneously. Two-temperature cycling is widely used in both legacy and faster PCR protocols. However, particularly in the case of longer amplicons, cycling speed can be increased if the extension temperature is optimal for enzyme activity. This can be achieved by either increasing the primer Tms so that annealing occurs at the optimal extension temperature, or modifying the instrumentation to provide three separate temperature targets that can be optimized individually. We now report a three-temperature extreme system and initial kinetic tests to define the temporal requirements at each stage.

## 2 Materials and methods

**Fig. 1:**
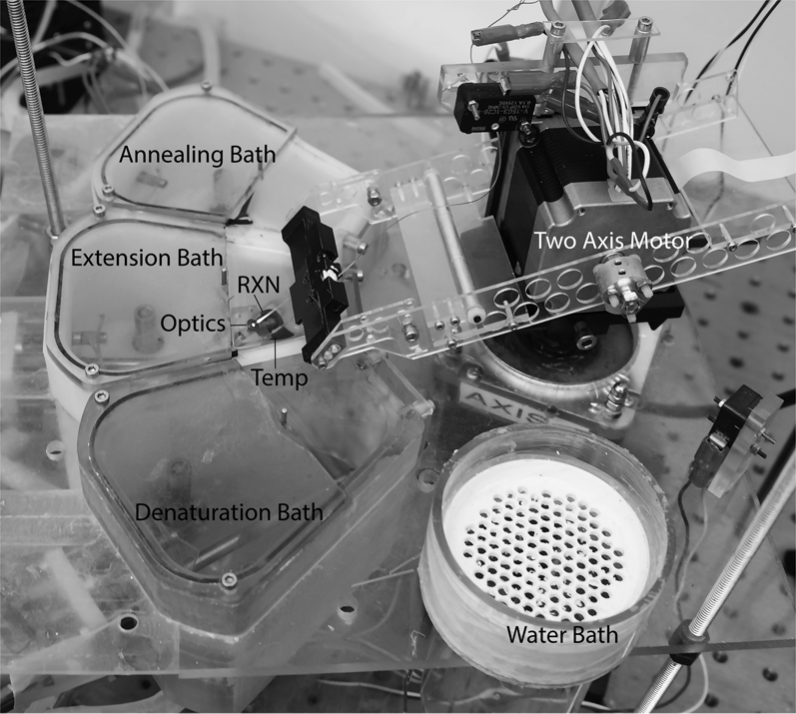
Extreme PCR instrument for this work. Two stepper motors, one attached atop the other, provide radial and vertical motion of an arm that holds two capillary tubes. One contains a miniature thermocouple immersed in 5 μL of water (Temp). The other capillary contains 5 μL of PCR solution (RXN) including the DNA dye, LCGreen Plus. When in the extension bath, the PCR capillary is aligned with a fiber optic for real time data acquisition by epifluorescence. Three water baths are used for primer annealing, polymerase extension, and DNA denaturation. A fourth bath containing ice water is used to hold capillaries in an inactive state before cycling. The instrument resembles the two-bath system previously reported [6]. Photo credit: LZ Photography.

Rapid cycle PCR was performed on a commercial capillary LightCycler [9,10]. Extreme PCR was performed on a second generation water bath system similar to that previously reported [6] with several improvements (Fig. 1). Instead of only two water baths, three cycling water baths and one additional bath for holding reactions in ice water before PCR were included. The single stepper motor that flipped capillaries between two baths was replaced with two stepper motors (vertical and horizontal) that translated and dipped the capillaries along a spherical shell between each bath. Epifluorescence through a fiber optic aligned and coaxial to the reaction capillary tip provided real-time fluorescence measurements. Sample temperature measurements were continuously recorded by a thin thermocouple placed in a mock sample capillary next to the reaction capillary. LabView (National Instruments) was used for actuator control and data acquisition.

### 2.1 Oligonucleotides

Phosphoramidite chemistry (Integrated DNA Technologies) was used to synthesize PCR primers. For extension studies, primers amplifying 100–800 bp products of 50% GC content based on lambda DNA were used [11,12]. Primers for denaturation studies amplified a 60 bp genomic fragment of *AKAP10* as previously reported [6]. For annealing studies, a 75 bp genomic product surrounding rs#11078849 was amplified using primers: ACCCTTGAAATAAAAGCTAATATTACTACCT and CAAATGTTTGGAATTTCTCAAAGATTTAATATTATATAAAA.

Templates were purified human genomic DNA for the annealing and denaturation studies. For the extension studies, G-blocks (IDT) for the 100–600 bp products or plasmids for the 700–800 bp products were used [11]. All oligonucleotides and templates were quantified by ultraviolet absorbance at A_260_ (Nanodrop, Thermo Fisher).

### 2.2 Polymerase chain reaction (PCR)

Rapid cycle PCR reagents were used for LightCycler PCR. Reactions of 10 μL were performed in 50 mM Tris, pH 8.3 at 25 °C (Sigma), 500 μg/ml BSA (Sigma), 200 μmol/L each dNTP (Bioline), 3 mmol/L MgCl_2_, 1X LCGreen Plus (BioFire), 0.04 U/μL (0.064 pmol/μL) of a deletion mutant of Taq polymerase (KlenTaq, DNA Polymerase Technologies), 12.8 ng/μL anti-Taq antibody (eEnzyme), 0.4 μmol/L each primer, and 1500 copies of G-block DNA. Annealing and denaturation experiments used the same reagents except that anti-Taq antibody was omitted and 5 μM primers, 1.6 μM KlenTaq, and 1500 copies of human genomic DNA were used in 5 μL reactions.

**Fig. 2:**
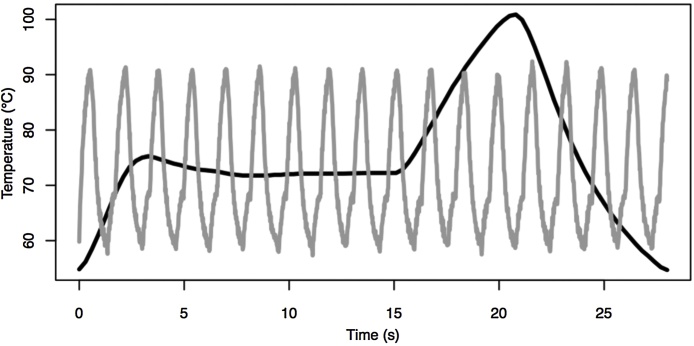
Actual sample temperatures for 3 temperature cycling obtained on a capillary LightCycler (dark grey) and the extreme temperature cycler (light grey). One cycle on the LightCycler takes just less than 30 s (< 15 min for 30 cycles). Thirty cycles on the extreme instrument takes about 50 s, about 18 times faster.

Temperature and time parameters on the LightCycler were 20 s of an initial denaturation, followed by 40 cycles of 95 °C for 0 s, 58 °C for 0 s, and 72 °C for 0, 2, 4, 6, 8, 10 or 12 s. Cqs were calculated by the second derivative method on LightCycler software. Fig. 2 compares the temperature trace for 1 cycle on the LightCycler with a 12 s extension to 18 cycles on the extreme instrument with no temperature holds.

**Fig. 3:**
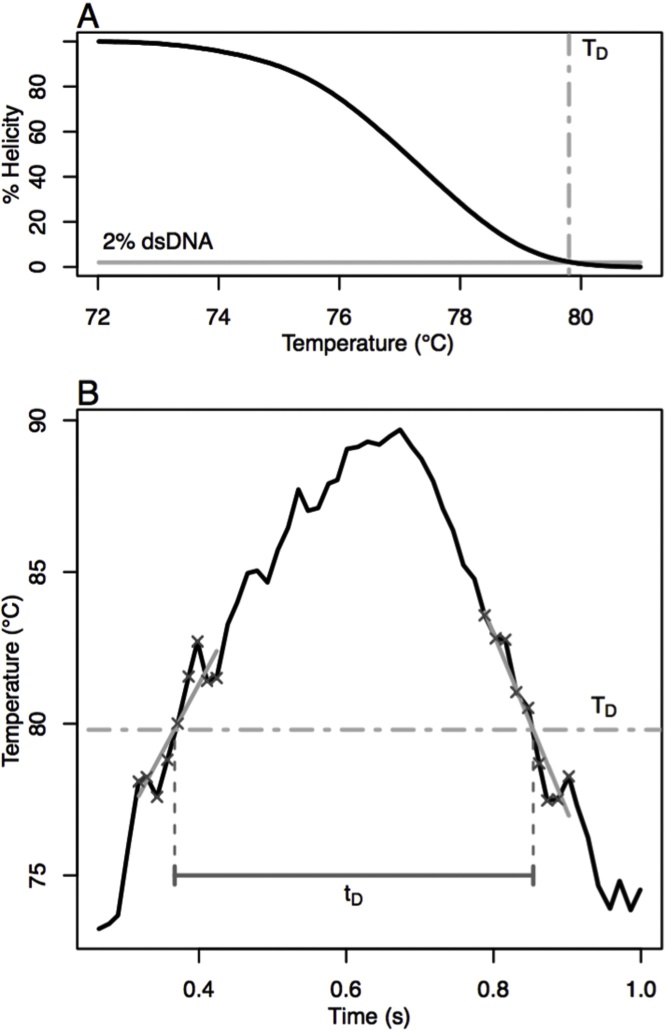
Description of the method for determining the denaturation threshold and measuring the denaturation time. Panel A shows the melting curve of the AKAP10 product after background subtraction and normalization. The denaturation temperature threshold (T_D_) was defined as the temperature at which the amplicon is 98% denatured. Panel B shows the temperature trace of the denaturation step of one typical extreme PCR cycle. The denaturation time (t_D_) was defined as the recorded time above T_D_. Each crossing point was taken as the intersection of T_D_ and a linear fit (gray line) of the 9 experimental data points (crosses) nearest the threshold.

For denaturation and annealing studies on the extreme cycler, only one stage was time limited. Under the conditions used, times of 1 s were excessive for denaturation, annealing, and extension. Therefore, to measure required denaturation times, annealing and extension were held for 1 s while denaturation times were varied above a denaturation threshold. The threshold denaturation temperature was defined as the temperature at which 98% of the product was denatured at equilibrium under PCR conditions. This temperature threshold was obtained by melting the product at 0.3 °C/s on a reference high resolution melting instrument (HR-1, BioFire). Similarly, a threshold annealing temperature was defined as the temperature at which 98% of the primer was annealed to its complement, the complement having 2 extra 5′ bases. Annealing and denaturation times were calculated from the actual temperature vs time curves measured during cycling. The denaturation time was the time the sample was at or above the threshold denaturation temperature, and the annealing time was the time the sample was at or below the threshold annealing temperature (Fig. 3). For the products used here, the threshold denaturation temperature was 79.8 °C with a product Tm of 77.3 °C. The threshold annealing temperature of the lowest Tm primer was 55.8 °C. Initial estimates for programmed temperature and times were empirically derived from trial runs. However, the actual times at or beyond the threshold temperatures were computed from the recorded data and correlated to Cq.

## 3 Results

**Fig. 4:**
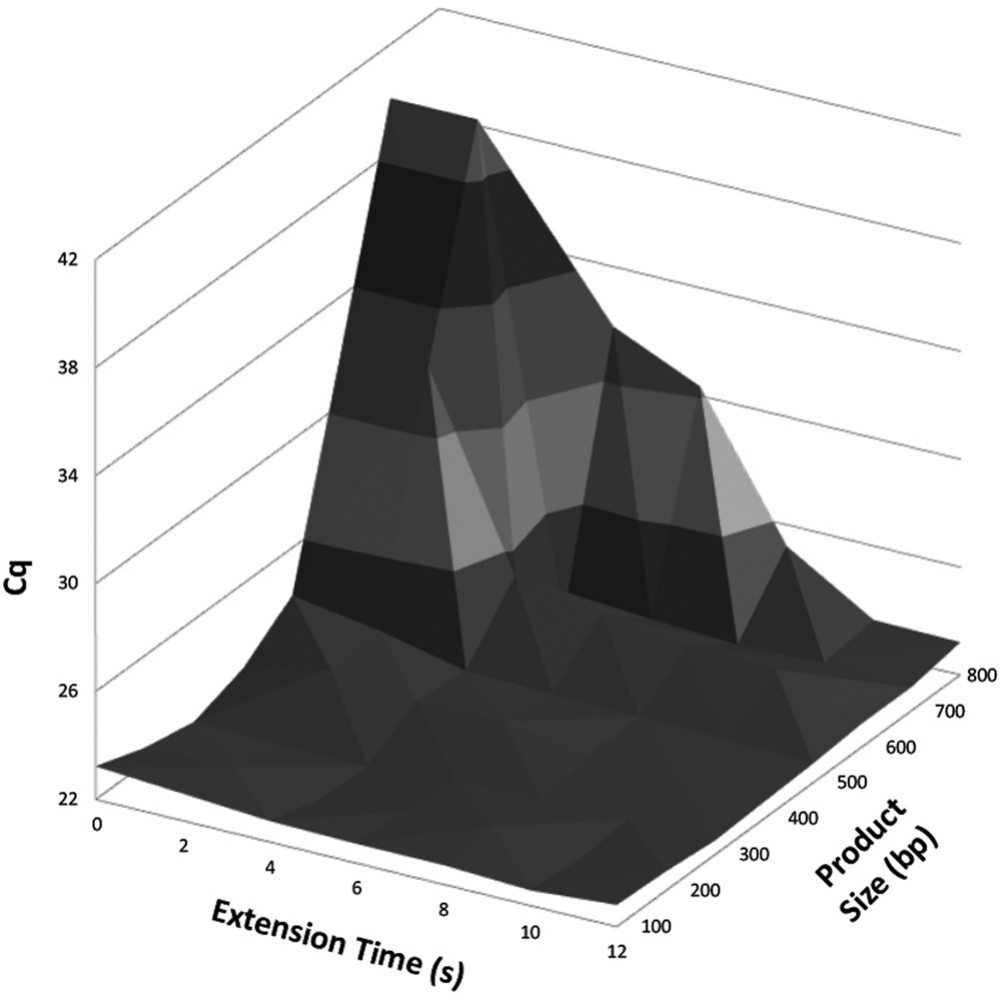
3D plot relating extension time and product size to Cq in real-time PCR performed on a capillary LightCycler. Short products (100–200 bps) amplified efficiently even without any hold during extension. However, longer products amplified poorly unless the extension time was increased. Points displayed with a Cq of 40 did not amplify after 40 cycles.

Polymerase extension times were studied by rapid cycle PCR on a commercial capillary LightCycler. Using primers that amplified 100–800 bp products, we varied the extension times from 0 to 12 s and measured resulting Cq values. As expected, larger products required more time for efficient amplification (Fig. 4). Products 100–200 bp in length amplified just as efficiently at “0” s with no hold as with longer times—that is, no holding time was necessary and extension was complete during the transition between annealing and denaturation. However, Cqs increased as the product size increased, unless more time was allowed. At 700–800 bp, 10–12 s were required, suggesting experimental polymerization rates during PCR of about 70 bases/s.

**Fig. 5:**
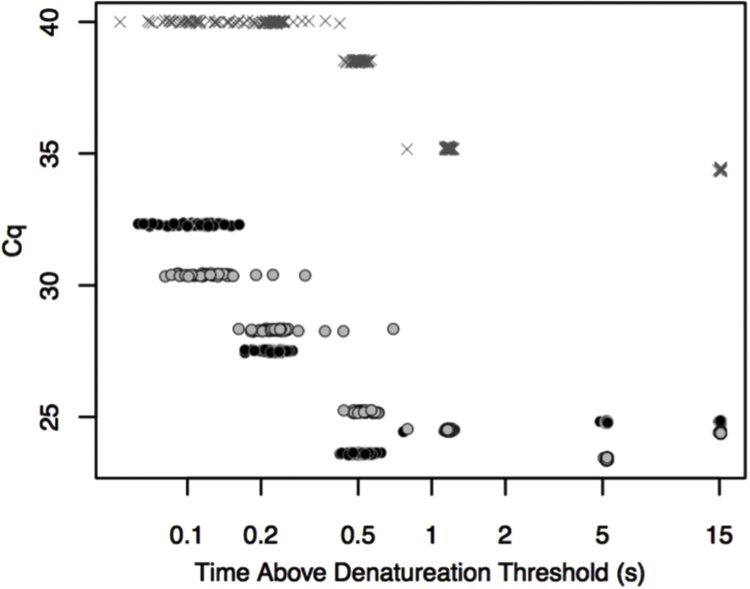
The effect of measured denaturation times on quantification cycle (Cq) using the extreme cycler. Programmed temperatures and times were empirically derived from trial runs such that the reaction was held above T_D_ for approximately 15, 5, 1, 0.5, 0.2, and 0.l s. For time points less than 1 s, the stepper motors were triggered to move the reaction capillary out of the denaturation bath once the thermocouple measurement reached a specified temperature. Lower temperature trigger points corresponded to shorter times spent above the denaturation threshold. For times of 1 s and longer, the motor was triggered after the controlling computer’s clock reached the specified time. Times reported in the figure are calculated post run in the manner discussed in Fig. 3. Each circle represents the time above the denaturation threshold for one cycle. Clusters of 40 circles represent all 40 cycles of 1 reaction. All 40 cycles are shown to indicate that there is still a level of variability that, particularly at shorter time scales, could have an effect on the overall reaction. Duplicates are denoted by black and gray circles along with selected no template controls (crosses). Some amplification of the negative controls is apparent at longer times, but the Cqs are about 10 greater than the positive runs.

The required denaturation time for one short PCR product is investigated in Fig. 5. Extension and annealing times were kept long so as not to limit amplification. The denaturation time was reduced until Cqs increased, indicating a loss of PCR efficiency. At denaturation times between 10 s down to 0.5 s, the Cq is steady between 24 and 25 cycles. At 200 ms however, the Cq increases to about 28, followed by further increases as the denaturation time is further reduced. This suggests that the required denaturation time to maintain maximal efficiency under these conditions is between 200 and 500 ms.

**Fig. 6:**
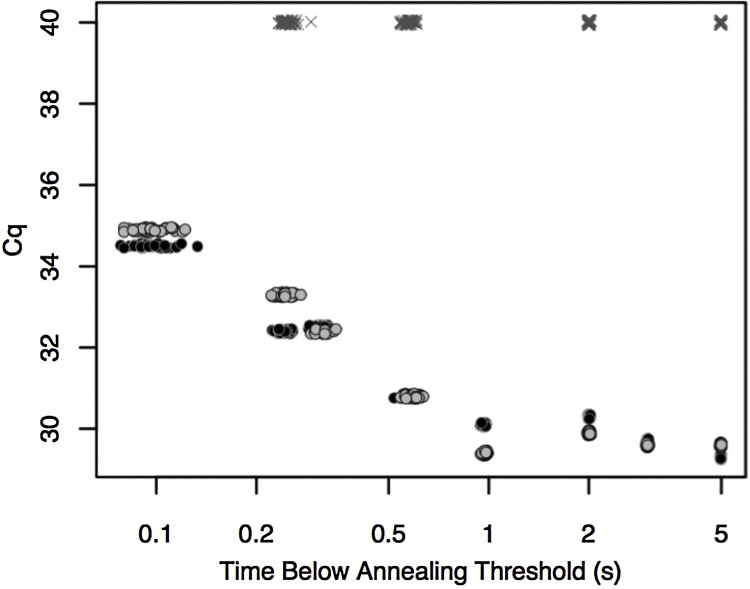
The effect of measured annealing times on Cq using the extreme cycler. Similar to Fig. 5, each cluster represents 40 determinations of times below the annealing temperature threshold, one for each cycle. Duplicate runs (black and gray circles) were performed at 5, 3, 2, 1, 0.6, 0.3, 0.2, and 0.l s, along with selected no template controls (crosses). No amplification of the negative controls is apparent after 40 cycles.

The required annealing time for another short PCR is investigated in Fig. 6. Denaturation and extension times were kept long so as not to limit amplification. The annealing time was reduced until Cqs increased, indicating a loss of PCR efficiency. At annealing times between 5 s down to 1 s, the Cq is steady around 30 cycles. At 600 ms however, the Cq increases slightly to 31, followed by 32–33 cycles at about 0.3 s and 35 at 0.1 s. This suggests that the required annealing time to maintain maximal efficiency under these conditions is between 300 and 1000 ms. Negative controls did not amplify after 40 cycles.

## 4 Discussion

In addition to its extensive utility in research and diagnostics, qPCR is also helpful in the study of PCR inhibitors and amplification efficiency [13]. When temperature or time parameters of PCR are optimal, efficiency is high and real-time quantification cycles (Cq) are low. Any increase in Cq reflects a compromise in reaction efficiency and deviation from optimal amplification conditions. Cq is used here to experimentally assess the time requirements for each stage of PCR.

Polymerase extension rates depend on many things, including the identity of the polymerase, buffer conditions, reactant concentrations, and temperature [14]. The amount of time required for extension in PCR is usually presumed to be proportional to the length of the product being amplified, although sequence effects on extension rates are also known [15]. Extension rates of approximately 60 bases/s at 70 °C for Taq polymerase [16] and 100 bases/s at 70 °C for KlenTaq [17] have been reported. Full length Taq is relatively slow compared to faster polymerases such as the deletion mutant KlenTaq or KAPA2G Fast [14]. Results here support these rates as limiting under PCR conditions with about 1 s required for every 70 bases using KlenTaq.

Compared to polymerase extension, product denaturation and primer annealing appear to be fast, with experimental evidence suggesting that <1 s is required for each stage [3]. DNA denaturation is a first-order reaction, usually carried out at temperatures high enough that the reverse reaction (hybridization) is not significant. When a denaturation temperature threshold is defined as the temperature at which 98% of the PCR product is denatured, 200–500 ms were required above the threshold for adequate denaturation (Fig. 5). An increase in Cq at 200 ms indicated a loss of PCR efficiency caused by incomplete denaturation.

Practical uses of denaturation times <1 s are limited by available instrumentation. Legacy protocols with long hold times reflect the sluggish temperature control and current limitations of most available instruments. It is hard to homogeneously change the temperature of aqueous samples quickly. Indeed, inadequate denaturation is perhaps the most common cause of PCR failure. Different samples may reach different maximal temperatures within the same instrument, resulting in different denaturation efficiencies [18]. The accuracy of qPCR can also be compromised by target melting behavior. Regions of high GC content are difficult to denature and thermodynamically ultra-fastened regions in human genomic DNA have been reported to compromise qPCR [19]. Strand separation is not complete until the highest melting domain of a target separates and it is wise to predict PCR product melting behavior by programs such as uMelt [20]. Nevertheless, complete denaturation in PCR can occur in <1 s as shown here if the temperature is accurately and rapidly controlled.

Primer annealing is a second order reaction, dependent on concentrations of both the primer and available single stranded product. During most of PCR, the primer concentration is in vast excess over the product and the reaction is pseudo first order, making the reaction progress at each cycle directly proportional to the primer concentration. Required annealing times can be shortened by increasing the primer concentrations as shown by extreme PCR [6]. With the high primer concentrations used here, 300 - 1000 ms was required for annealing. In this case, the annealing temperature threshold was chosen as the temperature at which 98% of primer is bound to its template. High primer concentrations can result in unintended amplification and primer dimers, but this tendency is controlled for here by the short annealing times.

**Fig. 7:**
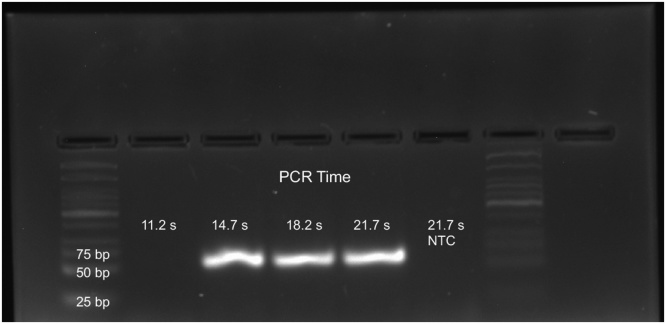
Two-temperature extreme PCR in less than 15 s. A 60 bp fragment of *AKAP10* was amplified from genomic DNA in 1 μL volumes. Thirty-five cycles were performed with 5 mmol/L MgCl_2_, 20 μmol/L each primer and 8 μmol/L polymerase. The minimum cycle time for amplification was 0.42 s, demonstrating that a specific, high yield of a 60 bp product can be obtained in under 15 s (35 cycles in 14.7 s). Differences from the current work include 2 vs 3 temperature cycling, and higher primer, polymerase, and MgCl_2_ concentrations. NTC = no template control. Reproduced from reference [6] (supplemental data) with permission of AACC.

The fastest extreme PCR protocol reported to date (with 0.42 s cycle times) was published as Supplemental Data in a Clinical Chemistry article [6] and is reproduced here as Fig. 7. Specificity and yield were excellent. However, the cycle time of 0.42 s is less than the sum of the minimum denaturation time of 0.2 s (Fig. 5) and minimum annealing time of 0.3 s (Fig. 6) determined here. How is this possible? Several experimental conditions enabled faster cycling in the prior work, including two vs three-temperature cycling, higher polymerase and primer concentrations, better heat transfer (with a metal capillary), a smaller temperature spread between denaturation and annealing, and higher Mg^++^ concentrations. Indeed, the products shown in Fig. 7 were amplified with 5 mM Mg^++^ and no amplification was observed at lower Mg^++^ concentrations [6]. Divalent cations are much more effective than monovalent cations in promoting hybridization [21].

When PCR was first developed, heat-stable polymerases and primer oligonucleotides were in short supply. This, combined with the ready availability and convenience of cycling heat blocks that were necessarily slow, resulted in long cycling times compatible with low concentrations of polymerase and primers to maintain adequate specificity. This historical precedent was convenient at the time but is not the only solution. The same specificity so critical to PCR is possible with high concentrations of primers and polymerases if the cycling times are vastly reduced. PCR does not have to be slow if temperature homogeneity is maintained and primer and polymerase concentrations are increased to speed the annealing and extension reactions [6]. Highly specific, sensitive, efficient and high yield PCR can be performed in less than 15 s (Fig. 7).

The feasibility of extreme PCR in 15 s, combined with rapid analytical tools such as high speed melting analysis that can differentiate between single nucleotide variants in 1 s [22] provide interesting diagnostic opportunities. Isothermal methods are not this fast and it’s presently difficult to imagine massively parallel sequencing at the beside. If targeted molecular questions, such as those raised by syndromic testing [23] can be answered in seconds or even a few minutes, diagnostics will be changed forever. Analytical speed is crucial to the value of point-of-care testing. Patients, physicians, and hospitals would welcome such testing if it can be performed at a reasonable cost.

## Conflict of interest statement

Aspects of extreme PCR are covered by pending and issued patents owned by the University of Utah and licensed to BioFire Diagnostics, a bioMerieux company. CTW may receive royalties for this technology through the University of Utah.
